# Bacteriological Profile and Antibiotic Susceptibility Pattern of Diabetic Foot Infection in a Tertiary Care Hospital in Lampung, Indonesia

**DOI:** 10.21315/mjms2021.28.5.4

**Published:** 2021-10-26

**Authors:** Iswandi Darwis, Hidayat Hidayat, Gusti Ngurah P Pradnya Wisnu, Sekar Mentari

**Affiliations:** 1 Department of Internal Medicine, Universitas Lampung, Lampung, Indonesia; 2 Department of Clinical Pathology, Dr Hi Abdul Moeloek Regional General Hospital, Lampung, Indonesia; 3 Department of Internal Medicine, Dr Hi Abdul Moeloek Regional General Hospital, Lampung, Indonesia

**Keywords:** diabetic foot, infection, microbiology, ulcer, antibiotic treatment

## Abstract

**Background:**

Diabetic foot infection (DFI) is a serious complication of diabetes mellitus and identification of the causative bacteria is an essential step in selecting the appropriate antibiotic therapy. This study aimed to evaluate the bacterial pattern and antibiotic susceptibility of the bacteria causing DFI in Lampung Province in Indonesia.

**Methods:**

This study is a retrospective study reviewing the medical records of DFI patients admitted to the Dr Hi Abdul Moeloek Regional General Hospital in 2017–2019. DFI patients with complete medical record data were included in this study. Demographic, clinical, laboratory, wound culture and antibiotic susceptibility data were collected from the medical records using a short structural chart. The data obtained then reviewed.

**Results:**

In this study, 131 DFI patients met the study criteria and were included. Based on the wound culture results, Gram-negative bacteria were obtained in 112 (85.5%) subjects with *Enterobacter* spp. as the predominant bacteria. Gram-positive bacteria were found in 19 (14.5%) subjects with *Staphylococcus* spp. as the predominant bacteria. Gram-negative bacteria found in this study showed high susceptibility to amikacin, meropenem and sulbactam/cefoperazone. Meanwhile, the Gram-positive bacteria showed high susceptibility to meropenem, sulbactam/cefoperazone and amikacin.

**Conclusion:**

The findings of the study revealed *Enterobacter* spp. as the most predominant bacteria causing DFI in the studied population. The highest antibiotic susceptibility was seen for amikacin, meropenem and sulbactam/cefoperazone.

## Introduction

Diabetes mellitus is one of the leading causes of morbidity and mortality in the world. To this day, an estimated 463 million adults live with diabetes worldwide. This figure is projected to grow to up to 700 million by 2045 (1). The 2012 Indonesia Basic Health Research (RISKESDAS) reported that the prevalence of diabetes in Indonesia was 2.1% in the population aged 15 and above (2).

Diabetes mellitus is an endocrine disorder that can result in various complications, including diabetic foot infection (DFI). The lifetime risk of Type 1 and Type 2 diabetes patients with DFI is 34% (3). Circulatory disorders due to peripheral arterial disease and diabetic peripheral neuropathy predispose people with diabetes to develop DFI. Infection occurring in DFI varies from simple superficial cellulitis to chronic osteomyelitis (4).

The bacteriological profile of DFI varies depending on the severity of the disease. *Staphylococcus aureus*, *Streptococcus pyogenes*, *Staphylococcus epidermidis*, *Escherichia coli*, *Pseudomonas aeruginosa*, *Klebsiella pneumonia*, *Acinetobacter* spp., *Proteus* spp. and *Enterococcus* spp. are the most common pathogens. The typical bacteriological profile also differs according to geographic location. Studies in temperate regions (North America and Europe) have consistently shown that the most common pathogens in DFI are aerobic Gram-positive cocci, particularly *Staphylococcus aureus* and coagulase-negative *Streptococcus* and *Staphylococcus*. Recent studies of DFI from tropical/subtropical regions (especially Asia and northern Africa) have shown that the main cause of DFI is aerobic Gram-negative bacilli, either alone or in combination with Gram-positive cocci. Obligate anaerobic bacteria can also play a role in DFI, especially in ischaemic limbs and DFI with abscesses (5).

The type of infection in DFI, either monomicrobial or polymicrobial, also depends on the severity of the disease. The severe form of DFI is usually caused by polymicrobial infections, whereas mild infections are frequently monomicrobial. In severe forms of DFI, up to three or five organisms may be cultured (5).

DFI is a complex complication of diabetes and is costly to treat. Apart from being a major cause of morbidity, DFI contributes to many hospitalisations and hospital admissions for people with diabetes and is the most frequent cause of nontraumatic proximal amputation. DFI is also associated with a risk of death of up to 2.5 times that of diabetes patients without DFI (3, 6).

Clinical guidelines from the Infectious Diseases Society of America (IDSA) recommend treating clinically infected diabetic foot ulcers with empirical antibiotics until microbiological culture results are available. Empirical antibiotics should be chosen in accordance with the severity of infection, clinical presentation and the prevalence of microorganisms in the local area and their antibiotic susceptibility. For DFI that occurs in diabetes patients in tropical/subtropical climates, IDSA recommends an empirical antibiotic regimen covering frequently isolated Gram-positive and Gram-negative pathogens as well as obligate anaerobes in moderate to severe DFI. The selection of subsequent antibiotic regimens must be carried out based on the clinical response, culture and antibiotic susceptibility results (5).

The variations in the bacteriological profile of DFI that depends on geographic, cultural and climatic factors could make the international clinical guidelines for the selection of appropriate empirical antibiotic therapy difficult to apply in developing countries (5, 7). This study was conducted to evaluate the bacterial profile and antibiotic susceptibility of bacteria causing DFI in people with diabetes in Lampung Province, Indonesia, to aid in the appropriate selection of empirical antibiotics.

## Methods

### Research Design

This is a retrospective study reviewing the medical records of diabetic patients with DFI treated at the Dr Hi Abdul Moeloek Regional General Hospital in 2017–2019. This hospital is a provincial hospital and the main referral hospital of Lampung Province. Patients with complications of diabetes are usually referred to this hospital. The study population was defined as the total number of patients with diabetes treated at the Dr Hi Abdul Moeloek Regional General Hospital. Sample sizes were calculated using the single proportion formula. Using the global prevalence of diabetic ulcers among diabetics (6.3%) (8) and taking a precision of 0.05, the calculated minimum sample size was 91. The level of confidence was set at 95%. The sampling technique used in this study was nonprobability with total sampling, a purposive sampling method, due to the limited population size of this study. The inclusion criteria for this study included: i) patients with DFI admitted to the Dr Hi Abdul Moeloek Regional General Hospital of Lampung Province; ii) patients with complete medical records including demographic data (gender and age), clinical data (length of hospital stay, in-hospital mortality), laboratory data (complete blood count and blood chemistry), wound culture and antibiotic susceptibility test results. Patients who had received systemic antibiotic therapy for more than 24 h in the 72 h prior to the collection of samples for microbiological and antibiotic susceptibility examinations were excluded from this study. Ethical clearance was obtained from the Medical Faculty of Universitas Lampung and the hospital authority before the commencement of this study.

### DFI Treatment Setting

DFI is defined clinically as the manifestation of an inflammatory process due to the invasion and multiplication of microorganisms in any tissue in a diabetic patient’s foot, the anatomical area under the malleoli. This condition usually results from disruption of the protective skin envelope at a site of trauma or ulceration in a patient with neuropathy or peripheral artery disease (5). We hospitalise DFI patients with moderate infection accompanied by limb ischaemia or a lack of response to outpatient treatment, severe infection, gangrenous tissue requiring amputation or the need for complex wound care. An endocrinologist or surgeon will take the lead as the physician in charge and treat the patients to increase the likelihood of limb salvage by combining medical treatment, revascularisation if needed and surgical management, which includes limited lower extremity amputation.

### Data Collection

The collection of medical records of diabetic patients with DFI was carried out through the Dr Hi Abdul Moeloek Regional General Hospital medical record database according to the 10th International Classification of Disease (ICD-10) diagnosis. The diagnosis of diabetes mellitus was defined as defective code E11 on the diagnosis of ICD-10. The diagnosis of DFI was confirmed by the presence of a diagnosis in the form of a handwritten medical record: ‘diabetic foot’, ‘diabetic foot ulcer’, ‘diabetic foot infection’ or ‘diabetic foot gangrene’. The data were then collected based on a questionnaire structured in several sections to collect various aspects of information from the medical record, including demographic data, history of diabetes, laboratory parameters during hospital stay, DFI bacteriological profile, antibiotic susceptibility profile, empirical antibiotic regimens given before the availability of microbiological culture results and susceptibility data, and observed outcomes in the form of mortality and length of hospital stay. The standard identification of the causative microorganisms in our hospital was carried out using standard microbiological methods with samples taken from the patient’s foot ulcer and antibiotic susceptibility testing was carried out using the disc diffusion method.

### Statistical Analysis

The data obtained from this study are summarised and then presented in diagrams, tables and bar charts as appropriate. Data analysis was performed using Statistical Package for Social Science software (IBM version 21.0; SPSS Inc., Chicago, IL, USA). Continuous variables are reported as mean (standard deviation [SD]) or median (interquartile range [IQR]) and categorical variables are reported as proportions. Distribution of microorganisms isolated in wound culture was reported as frequency (%), while antibiotic resistance and susceptibility were reported as proportion (%). Comparisons were conducted via Pearson’s chi-squared test or Fisher’s exact test for categorical variables and independent *t*-test or Mann-Whitney U test for continuous variables. The results were considered significant if the *P*-value was less than 0.05.

## Results

From 2017 to 2019, 131 patients were treated with DFI and met the inclusion criteria of this study. Among them, 57 (43.5%) were males and 74 (56.5%) were females. The mean age of the study subjects was 53.9 (9.2) years old. The mean length of stay of the subjects was 10.7 (5.9) days, with a median of 10 (IQR 8) days and 18 (13.7%) subjects died during hospital stay. The characteristics of the research subjects are shown in Table 1.

The patients’ mean haemoglobin was 9.3 (2.0) g/dL, with only 11 (8.4%) DFI patients without anaemia reported. The mean white blood count and platelet count were 19084.4 (7821.3)/mm^3^ and 422133 (170205)/mm^3^, respectively. The mean random blood glucose of the patient at admission was 265 (123.2) mg/dL.

The most widely used empirical antibiotic regimen for DFI treatment before antibiotic susceptibility data can be obtained in this study was a regimen consisting of ceftriaxone and metronidazole (56.5%), followed by ceftriaxone alone (15.3%), a regimen consisting of ciprofloxacin and metronidazole (14.5%), and other regimens (13.7%).

Based on the results of the wound culture, 13 pathogens were identified. Polymicrobial infection occurred among 10 (7.6%) subjects, while 111 (84.7%) others had a monomicrobial infection. The most common pathogens found were Gram-negative bacilli, with *Enterobacter* spp. (25.2%) as the predominant bacteria, followed by *Klebsiella* spp., *Proteus* spp., *Pseudomonas* spp., *Alcaligenes* spp., *Morganella* spp., *Sphingomonas* spp., *Escherecia* spp*., Acinetobacter* spp., *Citrobacter* spp. and *Yersinia* spp. Gram-positive cocci were also found with *Staphylococcus* spp. (10.7%) as the predmominant bacteria, followed by *Streptococcus* spp. These results are shown in Table 2.

There was no significant median length of stay difference between polymicrobial and monomicrobial infections among DFI (11.5 days versus 10 days; *P* = 0.204). Furthermore, mortality was revealed to not be associated with the nature of infection (*P* = 0.643) (shown in Table 3).

When the bacteria found in the wound culture of DFI patients were grouped based on Gram staining, it was found that Gram-negative bacteria were mostly susceptible to amikacin (95.5%), meropenem (92.6%) and sulbactam/cefoperazone (90.6%). Meanwhile, Gram-positive bacteria were mostly susceptible to meropenem (92.9%), sulbactam/cefoperazone (86.7%) and amikacin (80%). These results are shown in Tables 4 and 5.

The antibiotic susceptibility data revealed the most sensitive antibiotics for *Enterobacter* spp., *Klebsiella* spp. and *Pseudomonas* sp. as amikacin and for *Proteus* spp. as meropenem. Meanwhile, it was revealed that the most sensitive antibiotic for *Staphylococcus* spp. was sulbactam/cefoperazone (shown in Table 6).

The bacteria found in DFI were resistant to commonly used antibiotics. Gram-negative bacteria in this study showed the lowest susceptibility to chloramphenicol (28.3%), tetracycline (22.6%), cefixime (13.9%), ampicillin (13.4%), penicillin (11.1%), erythromycin (10.7%), amoxicillin (10.7%) and cefadroxil (10.6%). Meanwhile, Gram-positive bacteria showed the lowest susceptibility to chloramphenicol (30.8%), amoxicillin (23.1%), cefadroxil (21.4%), cefotaxime (11.1%), cefoperazone (10%), tetracycline (6.7%), cefixime (0%), clindamycin (0%), erythromycin (0%), ampicillin (0%) and penicillin (0%).

The individual components of empirical antibiotic regimens used in the Dr Hi Abdul Moeloek Regional General Hospital intended to cover aerobes bacteria that showed low susceptibility against Gram-negative or Gram-positive pathogens. Ceftriaxone showed only 34.1% and 50% susceptibility against Gram-negative and Gram-positive pathogens, respectively. Meanwhile, ciprofloxacin showed 52.2% and 37.5% susceptibility against Gram-negative and Gram-positive pathogens, respectively.

## Discussion

DFI is one of the main causes of morbidity in diabetic patients. It is more common in the older age group than in the younger groups. In this study, it was found that the mean age of patients with DFI was 53.9 years old. This is in accordance with the results of several other local studies that reported the average age of DFI patients to be over 50 years old (9–10). The majority of the patients with DFI in the present study were female. This contradicts previous studies that reported male predominance (9, 11–13). Poor glycaemic control, measured as HbA1c, fasting blood glucose or even single random blood glucose, has been shown to predict ulceration and subsequent amputation. In addition, it has also been reported to inhibit wound healing, making treatment even more difficult (14–15). In the present study, the mean of random blood glucose was 265 (123.2) mg/dL, above the acceptable limit for good glycaemic control.

Various types of bacteria can cause DFI and determining the specific bacteria causing this infection is an important step for a clinician to avoid using excessive and prolonged usage of broad-spectrum antibiotics. This aims to prevent various drug-related side effects, financial burdens, and antibiotic resistance (5).

Our study reported a predominance of monomicrobial infection in subjects of DFI, similar to several studies (12–13, 16–18). However, other studies have reported polymicrobial infection predominance (19–20). This discrepancy may be due to differences in clinical profiles or patient history (particularly the prior antibiotics scenario) of the study subjects (21). Another point to be noted here is that we did not consider anaerobic bacteria in our study.

The most common bacteria found in DFI in this study were Gram-negative bacilli, dominated by *Enterobacter* spp. and followed by *Klebsiella* spp., *Proteus* spp. and *Pseudomonas* spp. The present study confirmed Gram-negative bacilli as the most common bacteria causing DFI, as supported by several previous studies. Research by Pemayun and Naibaho (9) conducted at the Dr Kariadi General Hospital Medical Centre Semarang reported that Gram-negative bacillus was found in 70.8% of DFI cases. Another local study conducted by Bulolo et al. (10) at the Haji Adam Malik General Hospital Medan also reported that the most common bacteria found in DFI were Gram-negative bacilli with *Klebsiella pneumonia* (33.3%) as the predominant bacteria finding, followed by *Escherichia coli* (24.2%) and *Acinetobacter baumannii* (12.15%). Similar findings have previously been reported by various studies from other Southeast Asian countries (12–13, 22–24). The findings of the present study differ from the results of studies in America and European countries that reported Gram-positive bacteria as the main cause of DFI (5).

Apart from *Klebsiella* and *Escherichia coli*, other bacteria from Enterobacteriaceae families, such as *Proteus*, *Enterobacter* and *Citrobacter*, were also found in this study. The five of them live in the human digestive tract as normal flora (25). Environmental factors such as sanitation habits as well as the use of water to clean the perianal area after defecation, which causes hand contamination by the normal flora of faeces, are thought to affect the bacteriological profile of organisms causing DFI in developing countries, especially in rural areas (26).

Our findings reported no association between the nature of infection and length of hospital stay or in-hospital mortality. This finding contrasts with a study by Lipsky et al. (27), which reported cultures yielding polymicrobial *Pseudomonas aeruginosa* or monomicrobial Gram-negative (other than *Pseudomonas aeruginosa*) as independent risk factors for increased length of hospital stay and in-hospital mortality. However, our study did not address other factors that may contribute to the length of hospital stay and in-hospital mortality, such as severity of illness, transfer from another acute care hospital, surgical site infection or prior antibiotic use.

Diabetic patients with DFI have several factors that cause a high risk of carrying multidrug-resistant microorganisms (MDR), such as inappropriate previous administration of antibiotics, chronic wounds and frequent hospitalisations. The causative pathogen and antimicrobial susceptibility profile should be considered when selecting an antibiotic regimen (28).

This study found that Gram-negative bacteria were mostly susceptible to amikacin (95.5%). Amikacin is a semisynthetic kanamycin derivative from an aminoglycoside class antibiotic. Like other aminoglycosides, amikacin works by binding to the 30S subunit ribosomal protein, causing mRNA reading error. This, in turn, inhibits protein synthesis or causes defective protein production, which leads to the death of microorganisms. Amikacin is unique among aminoglycosides due to its resistance to aminoglycoside-inactivating enzymes, enabling its usage for various microorganisms that are usually resistant to other aminoglycosides. Amikacin and other aminoglycosides have an antibacterial spectrum covering a wide range of bacteria, including *Escherichia coli*, *Pseudomonas aeruginosa*, *Enterobacteria*, *Serratia*, *Proteus*, *Acinetobacter* and *Klebsiella*. Amikacin also shows antibacterial activity against *Staphylococcus* (29–30). The infrequent use of amikacin due to the high risk of ototoxicity and nephrotoxicity and the high price compared to other antibiotics may explain the fairly good susceptibility of various bacteria to this antibiotic (31). Apart from amikacin, Gram-negative bacteria also showed high susceptibility to meropenem (92.6%) and sulbactam/cefoperazone (90.6%). This is in line with a previous study by Kow et al. (12), which reported that Gram-negative bacteria in DFI showed high susceptibility to amikacin (100%) and meropenem (98.9%).

Meanwhile, most Gram-positive bacteria showed the highest susceptibility to meropenem (92.9%). Meropenem is an antibiotic from the carbapenem class with broad antibacterial activity against Gram-positive, Gram-negative and even anaerobic bacteria. Like other betalactam antibiotics, meropenem works by inhibiting the synthesis of bacterial cell walls, causing bacterial death. This antibiotic has excellent activity against *Staphylococcus aureus* and other methicillin-susceptible *Staphylococci* and most *Streptococcus* (32). Gram-positive bacteria also showed high susceptibility to sulbactam/cefoperazone (86.7%) and amikacin (80%).

In this study, the bacteria causing DFI showed resistance to commonly used antibiotics. Gram-negative bacteria showed the lowest susceptibility to chloramphenicol, tetracycline, cefixime, ampicillin, penicillin, erythromycin, amoxicillin and cefadroxil. Gram-positive bacteria showed the lowest susceptibility to chloramphenicol, amoxicillin, cefadroxil, cefotaxime, cefoperazone, tetracycline, cefixime, clindamycin, erythromycin, ampicillin and penicillin. Xie et al. (11) reported that Gram-negative bacteria, especially the Enterobacteriaceae family, exhibited a high degree of resistance to Ampicillin and antibiotics from the cephalosporin class. Xie et al. also reported that Gram-positive bacteria, especially *Staphylococcus aureus*, showed high resistance to commonly used antibiotics, such as penicillin and tetracyclines.

Individual components of empirical regimens given prior to the culture intended to cover aerobes in DFI were found to have low susceptibility against both Gram-negative and Gram-positive bacteria. The high rate of resistance to both the individual component of empirical antibiotic regimens given prior to the availability of causative pathogen identification and susceptibility data in the present study may be due to several factors, including hospitalisation history, recent use of broad-spectrum antibiotics, history of surgery and chronic wounds, irrational use of antibiotics and horizontal transfer of antibiotic resistance genes (18).

In the clinical setting, empirical antibiotic regimens are normally commenced to treat patients with DFI before the availability of microbiological culture results. They are intended to cover possible causative pathogens. The choice of empirical antibiotic regimens is often based on the patient’s clinical presentation and the bacteriologic profile of the local setting (12). The findings of this study can be used as a consideration in designing an empirical antibiotic treatment protocol for the management of DFI in the local setting.

Limitations of this study include the inability to generalise the study to the whole of Indonesia, as it was mainly a single-centre study. Due to the retrospective study design, pathogenic bacteria’s bacteriological profile differentiation based on the severity and grading of the DFI was not possible due to a lack of documented information in medical records. The lack of a specific culture for anaerobic bacteria is another limitation of this study. Further prospective studies are required to evaluate the clinical features and response to antibiotic treatment in DFI.

## Conclusion

This study showed that the most common organisms found in DFI were aerobic Gram-negative bacteria, with *Enterobacter* spp. being the predominant pathogen in the studied population. The bacteria found in DFI have varying resistance to commonly used antibiotics. This study’s findings provided valuable information concerning DFI in Lampung Province, Indonesia and might be used as a consideration for empirical antibiotic regimen selection to treat DFI in a local setting. Further studies with a larger sample size of the bacteria isolated from DFI in other areas in Indonesia are justified.

## Figures and Tables

**Table 1:** Characteristics of the study subjects

Characteristics	*n* = 131
Ages (years old)	53.9 (9.2)
Sex	
Male	57 (43.5%)
Female	74 (56.5%)
Length of stay(days)	10.7 (5.9)
Mortality	18 (13.7%)

**Laboratory data**	

Haemoglobin (g/dL)	9.3 (2.0)
WBC (/mm^3^)	19084.4 (7821.3)
Platelet (/mm^3^)	422133 (170205)
Random blood glucose (mg/dL)	265 (123.2)

**Table 2:** Bacteria isolated from a DFI

Bacteria	(*n*)	Percentage (%)
Gram-negative		
*Enterobacter* spp.	33	25.2
*Klebsiella* spp.	27	20.6
*Proteus* spp.	21	16.0
*Pseudomonas* spp.	17	12.9
*Alcaligenes* spp.	7	5.3
*Morganella* spp.	2	1.5
*Sphingomonas* spp.	2	1.5
*Escherecia* spp.	2	1.5
*Acinetobacter* spp.	1	0.7
*Citrobacter* spp.	1	0.7
*Yersinia* spp.	1	0.7
Gram-positive		
*Staphylococcus* spp.	14	10.7
*Streptococcus* spp.	3	2.3

**Table 3:** Mortality and length of hospital stay among patients with DFI

	Nature of infection		*P-*value

	Monomicrobial	Polymicrobial	
In-hospital mortality	16 (14.4%)	2 (20%)	0.643^a^
Length of hospital stay (days)	11.5 (7)	10 (8)	0.204^b^

Notes: Reported counts (proportions) for categorical variables and median (interquartile range) for continuous variables;

a Fisher’s exact test was applied;

b Mann-Whitney-U test was applied

**Table 4:** Antibiotic susceptibility pattern of Gram-negative bacteria isolated from a DFI

Antibiotics	Proportion of susceptibility (%)
Amikacin	95.5
Meropenem	92.6
Sulbactam/Cefoperazone	90.6
Ertapenem	75.9
Netilmicin	71.9
Piperacillin/Tazobactam	57.6
Trimethoprim/Sulfamethoxazole	57.1
Amoxicillin/Clavulanic	56.0
Piperacillin/Tazobactam	54.6
Gentamicin	53.0
Ciprofloxacin	52.2
Cefepime	51.5
Clindamycin	50.0
Sulbactam/Ampicillin	43.8
Ceftazidime	43.1
Cefoperazone	39.6
Ceftriaxone	34.1
Cefotaxime	30.9
Chloramphenicol	28.3
Tetracycline	22.6
Cefixime	13.9
Ampicillin	13.4
Penicillin	11.1
Erythromycin	10.7
Amoxicillin	10.7
Cefadroxil	10.6

**Table 5:** Antibiotic susceptibility pattern of Gram-positive bacteria isolated from a DFI

Antibiotics	Proportion of susceptibility (%)
Meropenem	92.9
Sulbactam/Cefoperazone	86.7
Amikacin	80.0
Trimethoprim/Sulfamethoxazole	66.7
Sulbactam/Ampicillin	66.7
Amoxicillin/Clavulanic	53.8
Ceftriaxone	50.0
Netilmicin	50.0
Ciprofloxacin	37.5
Gentamicin	33.3
Chloramphenicol	30.8
Amoxicillin	23.1
Cefadroxil	21.4
Cefotaxime	11.1
Cefoperazone	10.0
Tetracycline	6.7
Cefixime	0
Clindamycin	0
Erythromycin	0
Penicillin	0
Ampicillin	0

**Table 6:** Antibiotic susceptibility pattern of the five most commonly isolated bacteria from a DFI

Bacteria	Antibiotics	Proportion of susceptibility (%)
*Enterobacter* spp.	Amikacin	100
	Meropenem	100
	Sulbactam/Cefoperazone	87
	Trimethoprim/ Sulfamethoxazole	83.3
	Piperacillin/Tazobactam	80
	Ciprofloxacin	62.5
	Netilmicin	60
	Tigecycline	60
	Amoxicillin/Clavulanic	52.2
	Ceftazidime	50
*Klebsiella* spp.	Amikacin	100
	Sulbactam/Cefoperazone	100
	Meropenem	91.7
	Tigecycline	88.9
	Netilmicin	71.4
	Ertapenem	66.7
	Amoxicillin/Clavulanic	60
	Gentamicin	47.8
	Ceftriaxone	40
	Trimethoprim/Sulfamethoxazole	40
*Proteus* spp.	Meropenem	100
	Sulbactam/Cefoperazone	100
	Amikacin	80.9
	Ertapenem	75
	Piperacillin/Tazobactam	71.4
	Ciprofloxacin	63.6
	Cefepime	50
	Gentamicin	50
	Sulbactam/Ampicillin	50
	Trimethoprim/Sulfamethoxazole	50
	Ceftazidime	46.2
	Amoxicillin/Clavulanic	45.4
	Cefoperazone	45.4
*Pseudomonas* spp.	Amikacin	100
	Meropenem	100
	Sulbactam/Cefoperazone	88.9
	Cefepime	83.3
	Netilmicin	83.3
	Gentamicin	68.7
	Piperacillin/Tazobactam	66.7
	Amoxicillin/Clavulanic	50
	Trimethoprim/Sulfamethoxazole	50
	Ciprofloxacin	42.9
	Sulbactam/Ampicillin	42.9
	Ceftazidime	41.7
*Staphylococcus* spp.	Sulbactam/Cefoperazone	91.7
	Meropenem	90.9
	Amikacin	75
	Trimethoprim/Sulfamethoxazole	66.7
	Sulbactam/Ampicillin	66.7
	Ceftriaxone	50
	Amoxicillin/Clavulanic	50
	Netilmicin	50
	Chloramphenicol	40

## References

[1] International Diabetes Federation (2019). IDF Diabetes Atlas.

[2] Badan Penelitian dan Pengembangan Kesehatan (2013). Riset Kesehatan Dasar (RISKESDAS) 2013.

[3] Armstrong DG, Boulton AJM, Bus SA (2017). Diabetic foot ulcers and their recurrence. N Engl J Med.

[4] Amin N, Doupis J (2016). Diabetic foot disease: from the evaluation of the ‘foot at risk’ to the novel diabetic ulcer treatment modalities. World J Diabetes.

[5] Lipsky BA, Senneville E, Abbas ZG, Aragón-Sánchez J, Diggle M, Embil JM (2020). Guidelines on the diagnosis and treatment of foot infection in persons with diabetes (IWGDF 2019 update). Diabetes Metab Res Rev.

[6] Walsh JW, Hoffstad OJ, Sullivan MO, Margolis DJ (2016). Association of diabetic foot ulcer and death in a population-based cohort from the United Kingdom. Diabet Med.

[7] Uçkay I, Gariani K, Pataky Z, Lipsky BA (2013). Diabetic foot infections: state-of-the-art. Diabetes Obes Metab.

[8] Zhang P, Lu J, Jing Y, Tang S, Zhu D, Bi Y (2017). Global epidemiology of diabetic foot ulceration: a systematic review and meta-analysis. Ann Med.

[9] Pemayun TGD, Naibaho RM (2017). Clinical profile and outcome of diabetic foot ulcer, a view from tertiary care hospital in Semarang, Indonesia. Diabet Foot Ankle.

[10] Bulolo BA, Pase MA, Ginting F (2018). Antibiotic sensitivity pattern of bacteria from diabetic foot infections Haji Adam Malik central general hospital. IOP Conf Ser Earth Environ Sci.

[11] Xie X, Bao Y, Ni L, Liu D, Niu S, Lin H (2017). Bacterial profile and antibiotic resistance in patients with diabetic foot ulcer in Guangzhou, Southern China: focus on the differences among different Wagner’s grades, IDSA/IWGDF grades and ulcer types. Int J Endocrinol.

[12] Kow RY, Low CL, Ruben JK, Zaharul Azri WMZ, Japar Khan ESK (2019). Microbiology of diabetic foot infections in three district hospital in Malaysia and comparison with South east Asian countries. Med J Malaysia.

[13] Raja NS (2017). Microbiology of diabetic foot infections in a teaching hospital in Malaysia: a retrospective study of 194 cases. J Microbiol Immunol Infect.

[14] Dissanayake A, Vandal AC, Boyle V, Park D, Milne B, Grech R (2020). Does intensive glycaemic control promote healing in diabetic foot ulcers? a feasibility study. BMJ Open.

[15] Almaramhy H, Mahabbat NA, Fallatah KY, Al-Ahmadi BA, Al-Alawi HH, Guraya SY (2018). The correlation of fasting blood glucose levels with the severity of diabetic foot ulcers and the outcome of treatment strategies. Biomed Res.

[16] Hassan MA, Tamer TM, Rageh AA, Abou-Zeid AM, Abd El-Zaher EHF, Kenawy ER (2019). Insight into multidrug-resistant microorganisms from microbial infected diabetic foot ulcers. Diabetes Metab Syndr Clin Res Rev.

[17] Sánchez-Sánchez M, Cruz-Pulido WL, Bladinieres-Cámara E, Alcalá-Durán R, Rivera-Sánchez G, Bocanegra-García V (2017). Bacterial prevalence and antibiotic resistance in clinical isolates of diabetic foot ulcers in the Northeast of Tamaulipas. Mexico Int J Low Extrem Wounds.

[18] Anvarinejad M, Pouladfar G, Japoni A, Bolandparvaz S, Satiary Z, Abbasi P (2015). Isolation and antibiotic susceptibility of the microorganisms isolated from diabetic foot infections in Nemazee Hospital, Southern Iran. J Pathog.

[19] Saseedharan S, Sahu M, Chaddha R, Pathrose E, Bal A, Bhalekar P (2018). Epidemiology of diabetic foot infections in a reference tertiary hospital in India. Brazilian J Microbiol.

[20] Goh TC, Goh TC, Bajuri MY, Nadarajah S, Abdul Rashid AH, Baharuddin S (2020). Clinical and bacteriological profile of diabetic foot infections in a tertiary care. J Foot Ankle Res.

[21] Ahmadishooli A, Davoodian P, Shoja S, Ahmadishooli B, Dadvand H, Hamadiyan H (2020). Frequency and antimicrobial susceptibility patterns of diabetic foot infection of patients from Bandar Abbas District, Southern Iran. J Pathog.

[22] Abd Wahab NHH, Samsudin IN, Nordin SA, Ahmad Z, Noor LAM, Devnani AS (2015). Clinical presentation and microorganisms sensitivity profile for diabetic foot ulcers: a pilot study. Med J Malaysia.

[23] Vijaya-Kumar SL, Ashutosh SR, Gokulshankar S, Ranjith MS, Mohanty BK, Lim MY (2016). Is bacteriology a contributing factor in unsalvageable nature of diabetic foot infections? – a study in a district hospital in Malaysia. Int J Pharm.

[24] Abdul-Kadir KA, Satyavani M, Pande K (2012). Bacteriological study of diabetic foot infections. Brunei Int Med J.

[25] Rock C, Donnenberg MS, Caplan MJ (2014). Human pathogenic Enterobacteriaceae. Reference module in biomedical research.

[26] Suresh A, Muthu G, Srivani R, Moses A (2011). Aerobic bacterial resistance in diabetic foot ulcer from Chennai. Int J Pharm Bio Sci.

[27] Lipsky BA, Tabak YP, Johannes RS, Vo L, Hyde L, Weigelt JA (2010). Skin and soft tissue infections in hospitalised patients with diabetes: culture isolates and risk factors associated with mortality, length of stay and cost. Diabetologia.

[28] Ozer B, Kalaci A, Semerci E, Duran N, Davul S, Yanat AN (2010). Infections and aerobic bacterial pathogens in diabetic foot. African J Microbiol Res.

[29] Beauduy CE, Winston LG, Katzung BG, Masters SB, Trevor AJ (2017). Aminoglycosides and spectinomycin. Basic & clinical pharmacology.

[30] Ghazi IM, Grupper M, Nicolau DP (2017). Anti-staphylococcal activity resulting from epithelial lining fluid (ELF) concentrations of amikacin inhale administered via the pulmonary drug delivery system. Ann Clin Microbiol Antimicrob.

[31] El Zowalaty ME, Al Thani AA, Webster TJ, El Zowalaty AE, Schweizer HP, Nasrallah GK (2015). Pseudomonas aeruginosa: arsenal of resistance mechanisms, decades of changing resistance profiles, and future antimicrobial therapies. Future Microbiol.

[32] Fish DN (2006). Meropenem in the treatment of complicated skin and soft tissue infections. Ther Clin Risk Manag.

